# Functional analysis of epilepsy‐associated variants in STXBP1/Munc18‐1 using humanized *Caenorhabditis elegans*

**DOI:** 10.1111/epi.16464

**Published:** 2020-02-29

**Authors:** Bangfu Zhu, Jennifer C. H. Mak, Andrew P. Morris, Anthony G. Marson, Jeff W. Barclay, Graeme J. Sills, Alan Morgan

**Affiliations:** ^1^ Department of Cellular and Molecular Physiology Institute of Translational Medicine University of Liverpool Liverpool UK; ^2^ Max Planck Institute for Biology of Ageing Cologne Germany; ^3^ Department of Molecular and Clinical Pharmacology Institute of Translational Medicine University of Liverpool Liverpool UK; ^4^ Department of Biostatistics Institute of Translational Medicine University of Liverpool Liverpool UK; ^5^ Division of Musculoskeletal and Dermatological Sciences University of Manchester Manchester UK; ^6^ School of Life Sciences University of Glasgow Glasgow UK

**Keywords:** epileptic encephalopathy, exocytosis, nematode, SNARE, synapse

## Abstract

**Objective:**

Genetic variants in *STXBP1*, which encodes the conserved exocytosis protein Munc18‐1, are associated with a variety of infantile epilepsy syndromes. We aimed to develop an in vivo* Caenorhabditis elegans* model that could be used to test the pathogenicity of such variants in a cost‐effective manner.

**Methods:**

The CRISPR/Cas9 method was used to introduce a null mutation into the *unc‐18* gene (the *C. elegans* orthologue of *STXBP1*), thereby creating a paralyzed worm strain. We subsequently rescued this strain with transgenes encoding the human STXBP1/Munc18‐1 protein (wild‐type and eight different epilepsy‐associated missense variants). The resulting humanized worm strains were then analyzed via behavioral, electrophysiological, and biochemical approaches.

**Results:**

Transgenic expression of wild‐type human STXBP1 protein fully rescued locomotion in both solid and liquid media to the same level as the standard wild‐type worm strain, Bristol N2. Six variant strains (E59K, V84D, C180Y, R292H, L341P, R551C) exhibited impaired locomotion, whereas two (P335L, R406H) were no different from worms expressing wild‐type STXBP1. Electrophysiological recordings revealed that all eight variant strains displayed less frequent and more irregular pharyngeal pumping in comparison to wild‐type STXBP1‐expressing strains. Four strains (V84D, C180Y, R292H, P335L) exhibited pentylenetetrazol‐induced convulsions in an acute assay of seizure‐like activity, in contrast to worms expressing wild‐type STXBP1. No differences were seen between wild‐type and variant STXBP1 strains in terms of mRNA abundance. However, STXBP1 protein levels were reduced to 20%‐30% of wild‐type in all variants, suggesting that the mutations result in STXBP1 protein instability.

**Significance:**

The approach described here is a cost‐effective in vivo method for establishing the pathogenicity of genetic variants in STXBP1 and potentially other conserved neuronal proteins. Furthermore, the humanized strains we created could potentially be used in the future for high‐throughput drug screens to identify novel therapeutics.

**Key Points**

- Genetic variants in *STXBP1* are often associated with catastrophic infantile epilepsies
- Human wild‐type STXBP1 can functionally replace its worm orthologue, UNC‐18
- Expressing epilepsy‐associated human STXBP1 variants in worms causes functional impairments, including susceptibility to seizure‐like activity
- Humanized worms offer a cost‐effective approach to determining the pathogenicity of epilepsy‐associated variants in conserved genes

## 1 INTRODUCTION

Approximately one‐third of people with epilepsy do not respond to currently available treatments.^1^ This is likely due, at least in part, to a lack of knowledge of the underlying biological abnormalities that cause epilepsy and that determine individual responsiveness to medication. In recent years, genome‐wide association studies of epilepsy patients have begun to reveal genetic variants contributing effects to various forms of epilepsy. This could usher in a new era where therapies are tailored to the molecular genetic defects that give rise to individual epilepsies. However, achieving this goal requires genetically tractable animal models to study how specific epilepsy‐associated variants lead to functional impairments in vivo and to eventually translate this into therapies that can compensate for the genetic defect. The high financial costs associated with traditional rodent in vivo models of seizure and epilepsy have led to an increased awareness of the potential utility of simpler, non‐mammalian animal models for such studies over recent years.^2^

In 2008, a landmark study identified de novo mutations in *STXBP1* as the cause of Ohtahara syndrome (also known as early infantile epileptic encephalopathy with suppression‐burst) in five patients.^3^ Subsequent work confirmed this observation for Ohtahara syndrome^4^, ^5^ and extended it to patients with West syndrome,^6^ focal seizures with neonatal onset,^7^ and other early onset epileptic encephalopathies.^8^ More recently, an exome sequencing study of large numbers of patients with two early onset epileptic encephalopathies, infantile spasms and Lennox‐Gastaut syndrome, reported *STXBP1* to be the second most commonly mutated gene.^9^ More than 100 different *STXBP1* variants have been identified to date, including missense mutations, truncating mutations, and partial/whole gene deletions.^10^ It therefore appears that many cases of catastrophic infantile epilepsy are due to *STXBP1* mutations.

*STXBP1* encodes a neuronal member of the Sec1/Munc18 (SM) protein family^11^ known as Munc18‐1. SM proteins are essential for exocytosis in yeast (Sec1),^12^ *Caenorhabditis elegans* (UNC‐18),^13^ and mice (Munc18‐1),^14^ and their evolutionarily conserved function involves binding to membrane‐fusing SNARE proteins, notably syntaxin^15^ (hence the gene name: SynTaXin Binding Protein 1). Munc18‐1 can bind to isolated syntaxin in either its "closed" or "open" conformation and also binds to the synaptic SNARE complex comprising syntaxin/SNAP‐25/VAMP.^16^ Evidence that Munc18‐1 binding to syntaxin is relevant to epilepsy comes from the observation that knockin mice expressing a constitutively "open" mutant form of syntaxin 1B that is inefficiently sequestered by Munc18‐1 exhibit fulminant epilepsy.^17^ Furthermore, recent studies have reported that mutations in the gene encoding syntaxin 1B cause a range of different epilepsy syndromes in humans.^18^, ^19^ Hence, mutations in the intracellular machinery for synaptic vesicle exocytosis are emerging as a cause of epilepsy.

The number of genetic variants discovered in *STXBP1* associated with epilepsy and other neurological conditions is increasing rapidly due to dramatic improvements in DNA sequencing technologies.^10^ However, in silico methods for predicting whether individual variants are pathogenic often do not provide consistent conclusions for the same alteration.^20^ For example, the STXBP1/Munc18‐1 amino acid substitution variant E59K is predicted to be deleterious by Sorting Intolerant From Tolerant (SIFT) but is predicted to be benign by PolyPhen‐2.^10^ Work using cell culture models has provided important information on the functional impact of *STXBP1* haploinsufficiency and of several epilepsy‐associated missense variants in vitro.^3^, ^21^, ^22^, ^23^, ^24^, ^25^ Ideally, such studies would employ animal models to establish relevant functional impairments in vivo, as recently reported using null mutations in zebrafish (*stxbp1b*),^26^ haploinsufficiency in mice (*stxbp1*),^24^, ^27^ and missense mutations in *C. elegans* (*unc‐18*)^25^ gene homologues. Here, we have taken a different approach of creating humanized *C. elegans* strains by substituting expression of the endogenous worm UNC‐18 protein with transgenic expression of human Munc18‐1 protein (wild‐type or variant). We report our characterization of humanized worms harboring eight different epilepsy‐associated missense variants using a variety of functional analyses. Locomotion assays were chosen because they are simple to perform and amenable to automation and hence could potentially be used for future high‐throughput drug screens. An acute pentylenetetrazol (PTZ)‐induced seizure model was included as a further behavioral assay with relevance to epilepsy. Electrophysiological recording was employed to provide precise information about effects on neurotransmission in the pharyngeal nervous system. Finally, analyses of mRNA and protein expression were used to determine effects of STXBP1 variants on gene expression and protein stability.

## 2 MATERIALS AND METHODS

### 2.1 Materials

Plasmids used for genome modification via CRISPR/Cas9 were from Addgene. The original pMunc18‐1 plasmid^28^ was a generous gift from Dr Hitoshi Kitayama (Kyoto University, Japan). All other materials were from Sigma‐Aldrich unless stated otherwise.

### 2.2 Nematode culture

*C. elegans* were grown under standard conditions on nematode growth media (NGM; 2% [wt/vol] agar, 0.3% [wt/vol] NaCl, 0.25% [wt/vol] peptone, 1 mmol/L CaCl_2_, 5 μg/mL cholesterol, 25 mmol/L KH_2_PO_4_, 1 mmol/L MgSO_4_) agar plates. *Escherichia coli* OP50 was used as a food source. The wild‐type reference strain was Bristol N2. All strains were cultured and assays performed at 20°C. For drug treatment experiments, worms were cultured for 1 week (two‐three generations) on OP50‐seeded NGM agar plates containing either 5 mmol/L 4‐phenylbutyrate (Pb), 200 mmol/L sorbitol (Sb), or 200 mmol/L trehalose (Tr), as performed previously by Guiberson et al.^25^ Young adult worms were then subjected to locomotion assessment on freshly made unseeded NGM plates containing each chemical at the concentration described above.

### 2.3 Construction of unc‐18 null mutant strain

The *unc‐18(ulv12)* allele was generated using CRISPR/Cas9, following published procedures.^29^ The sgRNA sequence used to target *unc‐18* was 5′‐CGTTGACACCCTAGCCATGCGG‐3’. Wild‐type N2 animals were microinjected with a mixture of 25 ng/µL Cas9‐sgRNA plasmid, 50 ng/µL *sur‐5*::green fluorescent protein (GFP), and 10 ng/µL pMA122 (PEEL‐1 negative selection marker). Transgenic progeny was identified based on GFP expression and subsequently heat shocked at 34°C for 4 hours to induce PEEL‐1 expression, thereby killing animals carrying extrachromosomal arrays. Surviving worms that were nonfluorescent and paralyzed were selected as putative *unc‐18* mutants. Mutations were confirmed by single worm polymerase chain reaction (PCR) using *unc‐18* primers up‐ and downstream of the Cas9 site (forward primer: 5′‐GGGCACAGTAAGTTAGACGG‐3′; reverse primer: 5′‐GAGAAGAGCTGATCCGAACAC‐3′), followed by DNA sequencing (MRC PPU Centre, University of Dundee, Dundee, UK).

### 2.4 STXBP1 rescue strain construction

The original pMunc18‐1 plasmid,^28^ containing *STXBP1* cDNA under the control of the *C. elegans unc‐18* promoter and terminator elements, was first modified by site‐directed mutagenesis to correct an unintended ATG‐GTG mutation, which causes a predicted amino acid substitution, M316V. This new plasmid encoding sequence‐verified wild‐type *STXBP1* was then used as a template for further site‐directed mutagenesis to create the eight selected epilepsy‐associated missense mutations in Munc18‐1 (E59K, V84D, C180Y, R291H, P335L, L341P, R406H, and R3551C). Mutagenesis was performed using the Quick Change Site‐Directed Mutagenesis Kit (Stratagene). All mutations were confirmed by DNA sequencing.

Primers used for mutagenesis were E59K: 5′‐accgagggcatcacaattgtgaaggatatcaacaagcgccgagag‐3′, 5′‐ctctcggcgcttgttgatatccttcacaattgtgatgccctcggt‐3′; V84D: 5′‐atcaccccatctgagaagtctgaccactctctgatcagtgatttta‐3′, 5′‐taaaatcactgatcagagagtggtcagacttctcagatggggtgat‐3′; C180Y: 5′‐gcagagcagatcgcaaccctgtatgccaccctgaaggagtatcca‐3′, 5′‐tggatactccttcagggtggcatacagggttgcgatctgctctgc‐3′; R292H: 5′‐gatgacctgtggattgcgctgcatcacaagcacatcgcagaggtg‐3′, 5′‐cacctctgcgatgtgcttgtgatgcagcgcaatccacaggtcatc‐3′; P335L: 5′‐tcccagatgctgaagaaaatgctccagtaccagaaggagctcagc‐3′, 5′‐gctgagctccttctggtactggagcattttcttcagcatctggga‐3′; L341P: 5′‐atgccccagtaccagaaggagcccagcaagtattcgactcacctg‐3′, 5′‐caggtgagtcgaatacttgctgggctccttctggtactggggcat‐3′; R406H: 5′‐gtcagcacttacgacaaaatccatatcatccttctctacatcttc‐3′, 5′‐gaagatgtagagaaggatgatatggattttgtcgtaagtgctgac‐3′; R551C: 5′‐ggtgtgagcctgaatgagatgtgctgtgcttacgaagtgacccag‐3′, 5′‐ctgggtcacttcgtaagcacagcacatctcattcaggctcacacc‐3′.

### 2.5 Transformation of *C. elegans*

Germline transformation of *unc‐18(*ulv12*)* worm stains with DNA was performed by gonadal microinjection. *unc‐18(*ulv12*)* worms were injected with wild‐type or mutated pMunc‐18‐1 plasmids at 10 ng/μL along with a coinjection reporter construct, pTG96 (*sur‐5::GFP*) at 25 ng/μL, to identify transformed worms. All transgenic lines were confirmed by endpoint PCR using *STXBP1*‐specific primers (forward primer: 5′‐CAAGAGGATGAACACTGGCGAG‐3′; reverse primer: 5′‐CCATCGTGAGAGCTGGTAGGTCTG‐3′) and GoTaq DNA polymerase (Promega). At least three independent lines of each rescue strain (wild‐type and variant) were used for analysis.

### 2.6 Thrashing assay

Locomotion was measured in solution and quantified as thrashes per minute (one thrash defined as one complete sinusoidal movement from maximum to minimum amplitude and back again), as previously described.^30^ Single hermaphrodites were removed from NGM plates and placed in a Petri dish containing 200 μL freshly made Dent solution (140 mmol/L NaCl, 6 mmol/L KCl, 1 mmol/L CaCl_2_, 1 mmol/L MgCl_2_, 5 mmol/L hydroxyethylpiperazine ethane sulfonic acid, pH 7.4 with bovine serum albumin at 0.1 mg/mL). Assessment of locomotion was performed 10 minutes following immersion in solution. Ten animals per strain were analyzed in each experiment, and three independent experiments were performed (n = 30 worms per strain in total).

### 2.7 Body bend assay

Locomotion was quantified here as number of body bends per minute on freshly made unseeded NGM plates. A body bend was defined as one complete sinusoidal movement from maximum to minimum amplitude and back again (through the middle axis of the worm). Ten animals per strain were analyzed in each experiment, and three independent experiments were performed (n = 30 worms per strain in total).

### 2.8 Seizure assay

This was performed using a recently described method.^31^ Briefly, worms were exposed to 7 mg/mL PTZ in Dent solution for 15 minutes and then head‐bobbing convulsions were stringently scored over 30 seconds. Both the number of convulsions experienced per animal and the proportion of worms exhibiting seizure‐like activity were measured. The seizure‐prone *unc‐49(e407)* strain was used as a positive control and the seizure‐resistant wild‐type N2 strain as a negative control. Ten animals per strain were analyzed in each experiment, and three independent experiments were performed (n = 30 worms per strain in total).

### 2.9 Electropharyngeal recording

Electrophysiological extracellular recording was used to analyze pharyngeal neural and muscular activity. Electropharyngeograms (EPGs) were acquired with the NemaMetrix ScreenChip system, which uses microfluidic chambers to position the pharynx of individual worms close to recording electrodes. Young adult worms were collected and incubated in M9 buffer containing 10 mmol/L serotonin to stimulate pharyngeal pumping. Through adjusting the pump pressure, one worm was loaded into the channel in the center of the Screenchip between the recording electrodes using a dissecting microscope. Pharyngeal electrical activity was recorded for 3 minutes, with relevant parameters being monitored, such as mean pump frequency, pump amplitude, pump duration, interpump interval (IPI) duration, and standard deviation. EPG data were acquired and analyzed using NemAcquire and NemAnalysis software (NemaMetrix). Ten worms of each strain in total were recorded in these experiments.

### 2.10 Real‐time quantitative PCR

Total RNA was extracted from worm strains using an RNeasy Mini Kit (QIAGEN) according to the manufacturer's instructions. The total yield of RNA per extraction was measured by NanoDrop spectrophotometer (Thermo Fisher Scientific). cDNA was synthesized from 2000 ng RNA using MMLV reverse transcriptase (Promega) with Oligo(dT)s. PCR reactions were performed using iTaq Univer SYBR Green Super mix (Bio‐Rad Laboratories). *STXBP1*‐specific primers (forward primer: 5′‐CCCTATGAGTCCCAGGTGTATT‐3′; reverse primer: 5′‐GCGTTCCAGTATCGGATTCTTC‐3′) were used for target gene amplification, and the housekeeping gene *cdc‐42* was used for normalization, in keeping with published recommendations^32^ (*cdc‐42* forward primer: 5′‐GGTGGCGAGCCATACACATTAGG‐3′; reverse primer: 5′‐CTCTCCAACATCCGTTGACACTGG‐3′). The PCR reactions were run using a CFX Connect QPCR System (Bio‐Rad Laboratories) under the following cycling conditions: an initial denaturation step of 95°C for 3 minutes followed by 40 cycles of 10 seconds denaturation (95°C), 30 seconds annealing at 58°C, and 20 seconds elongation at 72°C. The relative amount or fold change of the target gene expression was normalized relative to the level of *cdc‐42* and relative to a control sample (*STXBP1* wild‐type rescue strain).

For quantitative PCR (qPCR) readings, three separate biological replicate cDNA samples were used, and each was measured in triplicate.

### 2.11 Western blotting

Worm strains were harvested from two 60‐mm‐diameter NGM plates in chilled M9 buffer and allowed to settle on ice for 10 minutes. After two washes in M9 buffer, the worm pellets were resuspended in Tris‐Glycine SDS Sample Buffer (Life Technologies). Protein concentrations were determined using a NanoDrop spectrophotometer (Thermo Fisher Scientific).

Samples of 50 µg total protein were separated by sodium dodecyl sulfate–polyacrylamide gel electrophoresis using precast 4%‐12% NuPAGE Bis‐Tris gels (Invitrogen) and then transferred to nitrocellulose membrane (PALL Life Sciences). The membrane was blocked overnight at 4°C in blocking solution (Tris‐buffered saline [TBS]: 20 mmol/L Tris (pH 7.4), 150 mM NaCl, with 0.1% Tween 20 [TBS with Tween (TBST)] and 5% [wt/vol] dried skimmed milk), and incubated at 4°C overnight with a primary antibody in TBST containing 1% bovine serum albumin. After three washes with TBST, the blots were incubated for 1 hour at room temperature with horseradish peroxidase–conjugated mouse or rabbit IgG secondary antibody (1:2000, Sigma‐Aldrich) and then washed three times with TBST. Detection was performed using Clarity Western ECL Substrate (Bio‐Rad Laboratories) according to the manufacturer's instructions and imaged in a Bio‐Rad ChemiDoc XRS using ImageLab software. The primary antibodies used were rabbit anti‐Munc18‐1 (STXBP1; Abcam, 1:1000), mouse anti‐GFP (Roche, 1:1000), and mouse anti–β‐actin (Sigma‐Aldrich, 1:2000). The relative protein expression levels were quantified by densitometry using ImageJ Gel Analysis software. Western blots from at least three independent biological replicate experiments for each worm strain were used for quantification.

### 2.12 Statistical analysis

Data are expressed as mean ± standard error of the mean. Phenotypic differences were determined using IBM SPSS Statistics 24. One‐way analysis of variance (ANOVA) with post hoc tests employing Tukey correction for multiple comparisons was used whenever normality and homoscedasticity criteria were met. Otherwise, nonparametric tests were performed by the Kruskal‐Wallis test with post hoc tests employing Dunnett correction for multiple comparisons. An error probability level of *P* < .05 was accepted as statistically significant and indicated with an asterisk throughout the study. Exact *P* values for each comparison are listed in the figure and table legends.

## 3 RESULTS

### 3.1 Creation of humanized *C. elegans* strains

**Figure 1:**
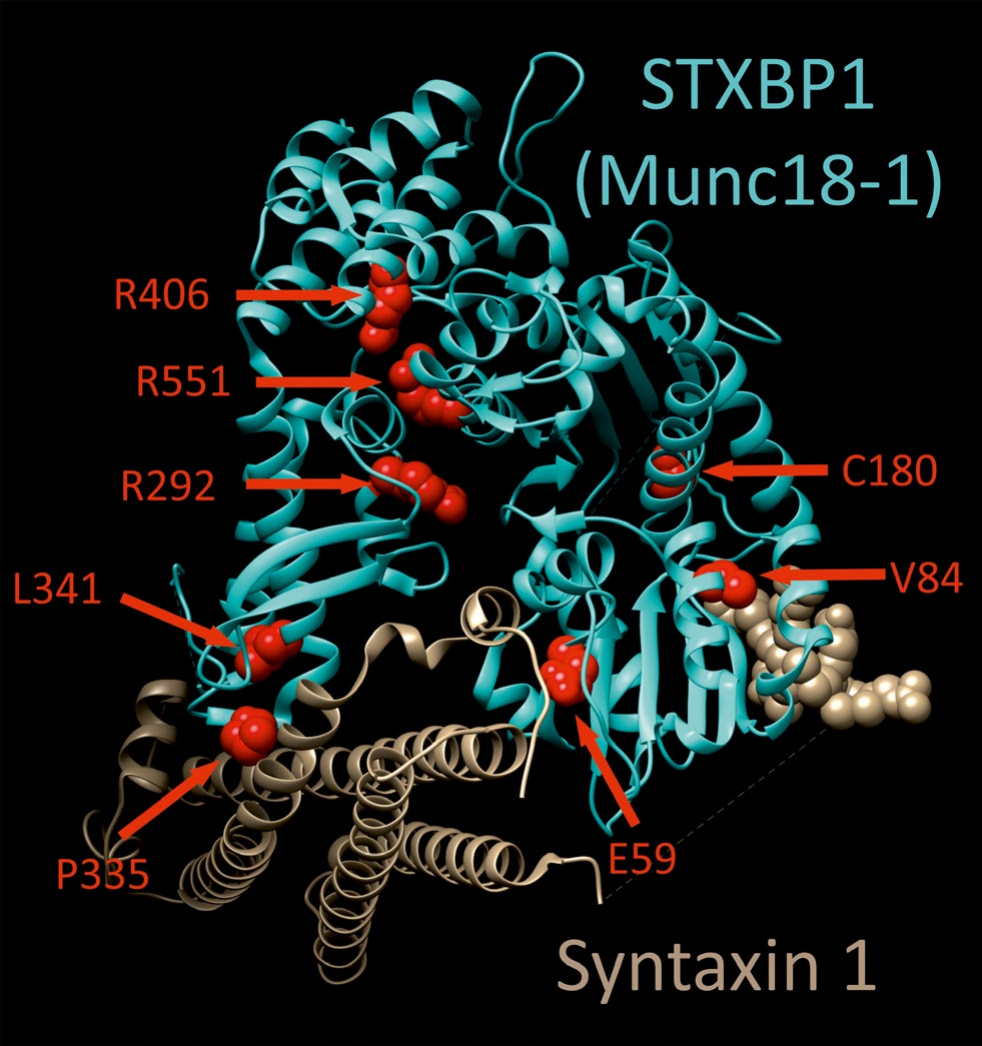
Human STXBP1 (Munc18‐1) protein structure and mutations. Crystal structure of STXBP1 (Munc18‐1) bound to syntaxin‐1 (Protein Data Bank code: 3C98) is shown, rendered using UCSF chimera. The positions of the 8 amino acids mutated in this study are highlighted as red spheres

To assess the functional effects of epilepsy‐associated *STXBP1* variants in a living animal, we adopted a knockout/rescue approach. First, we created a null mutant allele of the *C. elegans STXBP1* orthologue, *unc‐18*, using the CRISPR/Cas9 method.^29^ The *unc‐18(ulv12)* allele we generated contains a 7‐bp deletion that introduces a premature stop codon, which would truncate the predicted UNC‐18 protein after only 58 residues (Figure S1). This removes the domains of UNC‐18 that mediate binding to syntaxin (Figure 1A), an interaction that is essential for neurotransmission in *C. elegans.* 30, 33 Hence, even if the truncated mutant protein was expressed, it would be expected to be nonfunctional. Phenotypically, *unc‐18(ulv12)* worms were slow growing and virtually paralyzed (Video S1), similar to previously reported *unc‐18* null mutants such as the *e81* reference allele.^13^ We then set out to rescue the paralysis of these *unc‐18* mutant worms by expressing wild‐type or mutant human STXBP1/Munc18‐1 protein. The plasmid construct used contains mammalian cDNA flanked by the endogenous *C. elegans unc‐18* promoter and terminator elements,^34^ to ensure expression of Munc18‐1 protein in physiologically relevant worm cell types. Eight different epilepsy‐associated missense variants were chosen to reflect a range of different locations within the Munc18‐1 protein (Figure 1A). The reported incidence of these variants ranged from a single patient to the most highly recurrent (seven patients for R406H; Table 1). All are evolutionarily conserved in the worm UNC‐18 protein, suggesting an important function of each mutated amino acid (Figure S2). Mutations were introduced into *STXBP1* by site‐directed mutagenesis and microinjected into the gonads of *unc‐18(ulv12)* worms. Endpoint PCR confirmed that all strains had successfully incorporated *STXBP1* transgenes as extrachromosomal arrays (Figure S3), enabling analysis of the humanized knockout/rescue strains.

**Table 1:** Properties of STXBP1 variants analyzed in this study

Variant	Incidence	Conditions	References
E59K	1	NSE + ID	49
V84D	1	OS	3
C180Y	1	OS	3
R292H	5	EOEE; NSE + ID; WS	9, 10, 50
P335L	1	EOEE	9
L341P	1	EOEE	10
R406H	7	OS; EOEE; WS + RS	4, 9, 51, 52
R551C	4	EOEE	10, 53, 54

Note

Information on the number of patients affected and diagnosed conditions was taken from the ClinVar database and from Stamberger et al.^10^

Abbreviations: EOEE, early onset epileptic encephalopathy; ID, intellectual disability; NSE, nonsyndromic epilepsy; OS, Ohtahara syndrome; RS, Rett syndrome; WS, West syndrome.

### 3.2 Functional consequences of STXBP1/Munc18‐1 variants

Upon initial inspection of the humanized strains, it was obvious that wild‐type Munc18‐1 expression rescued the paralysis of the *unc‐18(ulv12)* strain (Video S2). These worms moved in a fully coordinated manner similar to that of the wild‐type N2 strain (Video S3). To quantify locomotion in the various worm strains, we measured the number of body bends produced over time as worms crawl over the surface of an agar plate.

**Figure 2:**
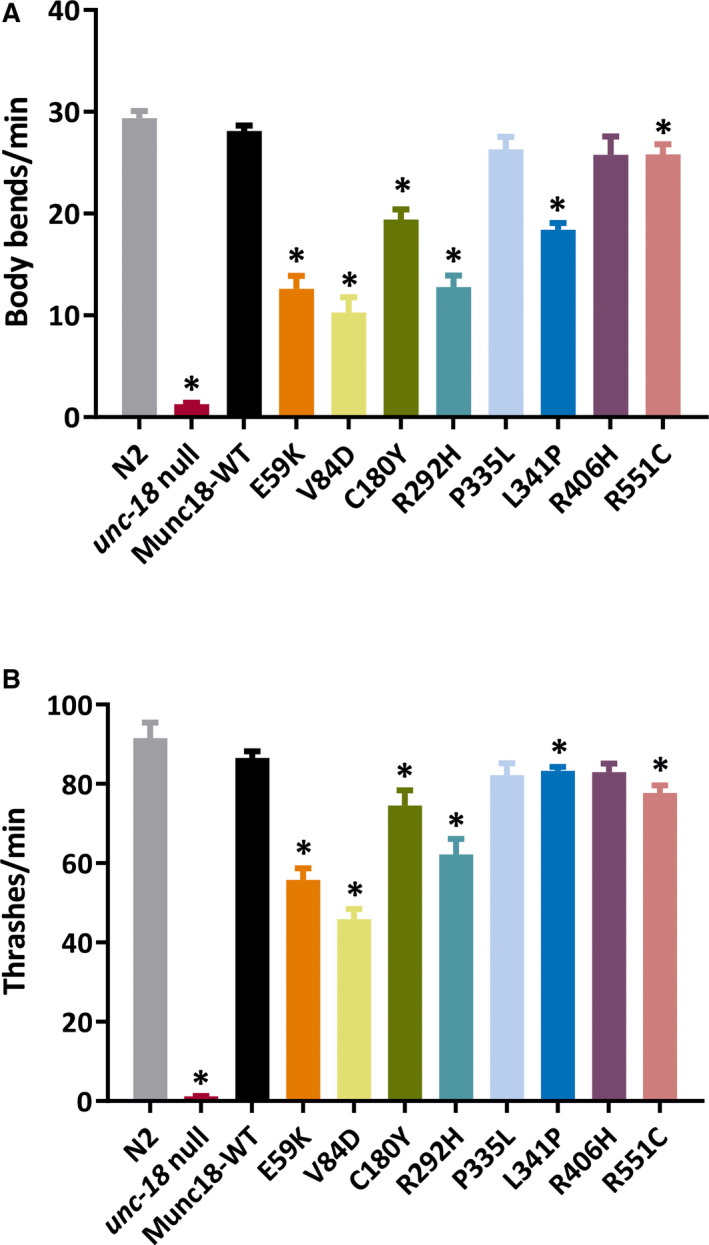
Behavioral analyses of STXBP1 transgenic worms. Locomotion was quantified either by counting the number of body bends executed per minute as worms crawled on an agar surface (A), or by counting the number of thrashes per minute as worms swam in solution (B). Six STXBP1 variants (E59K, V84D, C180Y, R292H, L341P, and R551C) showed significantly defective locomotion compared to worms expressing wild‐type STXBP1. The standard N2 wild‐type strain was included as a positive control and the paralyzed *unc‐18* null mutant strain as a negative control. Ten animals per strain were analyzed in each experiment, and three independent experiments were performed (n = 30 worms per strain in total). Data are shown as mean ± standard error of the mean. Statistical analysis was performed using one‐way analysis of variance with Tukey correction for multiple comparisons; *P < .05. Significant *P* values of STXBP1 mutants are as follows: A, E59K (.000041), V84D (.000044), C180Y (.000178), R292H (.000031), L341P (.000040), and R551C (.025338); B, E59K (.000094), V84D (.000022), C180Y (.007994), R292H (0.000605), L341P (.049196), and R551C (.004297)

Wild‐type Bristol N2 animals moved rapidly (around 30 body bends per minute), whereas *unc‐18(ulv12)* worms remained immobile for most of the assay period (Figure 2A). Transgenic expression of wild‐type Munc18‐1 protein restored locomotion in *unc‐18(ulv12)* worms to levels that were not significantly different from wild‐type N2 worms (Figure 2A), consistent with the initial visual inspection. Having confirmed that mammalian Munc18‐1 can functionally replace the natural *C. elegans* UNC‐18 protein,^28^ we then analyzed the effects of the eight epilepsy‐associated variants. Six of the transgenic strains (E59K, V84D, C180Y, R292H, L341P, R551C) exhibited significantly reduced body bends on agar compared to control worms expressing wild‐type Munc18‐1. The extent of this locomotion impairment ranged considerably between variants, however; for example, R551C retained 92% of wild‐type control Munc18‐1 locomotion activity, whereas V84D displayed only 37% activity (Figure 2A, Video S4). Two variants (P335L, R406H) were not significantly different from wild‐type control Munc18‐1 worms. We then used an alternative liquid‐based locomotion assay, to determine whether results might differ from those using solid media. When placed in liquid, *C. elegans* swim with a characteristic thrashing motion, enabling locomotion to be quantified by measuring the number of thrashes over time. The results in liquid media were generally similar to that seen on solid media; expression of wild‐type Munc18‐1 protein restored locomotion in *unc‐18(ulv12)* worms to N2 levels and the same 6 variants exhibited significantly reduced locomotion compared to control, albeit that the extent of inhibition in the variants was generally less marked in liquid (Figure 2B).

**Figure 3:**
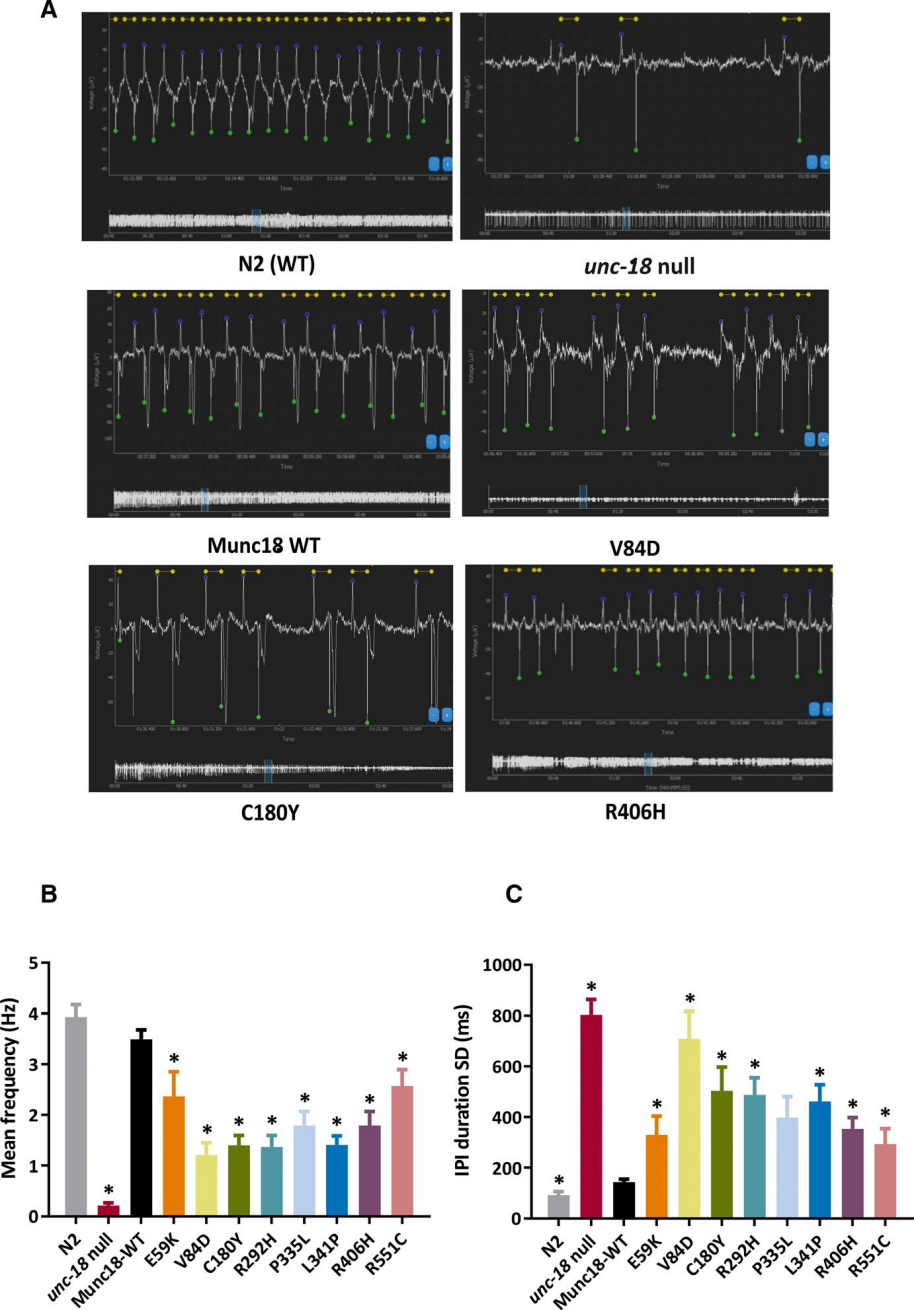
Electropharyngeographic (EPG) recordings from STXBP1 transgenic worms. A, Representative EPG recordings from N2 wild‐type (WT), Munc18 rescue, unc‐18 null, and three of the human STXBP1 variants, showing overall EPG recording information such as waveform, mean pump frequency, pump duration, and amplitude. B, All STXBP1 variants showed a significantly lower overall mean pump frequency compared to worms expressing WT STXBP1. C, The interpump interval (IPI) duration standard deviation (SD) represents the regularity of the pharyngeal pumping rhythm. All STXBP1 variants had a significant increase in IPI duration SD, indicating that pumping in the mutant strains is more arrhythmic than worms expressing WT STXBP1. Ten animals per strain were analyzed, and data are shown as mean ± standard error of the mean. Statistical analysis was performed using one‐way analysis of variance with Tukey correction for multiple comparisons (*P < .05; significant *P* values are shown in Figure S4)

We next used an electrophysiological approach to determine the effect of Munc18‐1 variants on neuromuscular transmission in the worm pharynx. Although pharyngeal muscle contraction has intrinsic myogenic capacity, the rhythmic high‐frequency contractions seen during feeding and in response to serotonin application require synaptic transmission via the pharyngeal nervous system.^35^, ^36^ Typical examples of EPGs from extracellular recordings of pharyngeal electrical activity are illustrated in Figure 3A. Wild‐type Bristol N2 worms exhibited consistently rhythmic pharyngeal pumping at a frequency of around 4 Hz in response to serotonin. In contrast, *unc‐18(ulv12)* worms displayed irregular and infrequent pharyngeal activity, as expected due to the severely reduced synaptic exocytosis caused by loss of UNC‐18 function. Expression of wild‐type Munc18‐1 protein rescued pharyngeal pumping frequency to levels that were not significantly different from N2 worms (Figure 3B), although pumping regularity was not quite restored to N2 levels, as indicated by the slightly higher IPI standard deviation value (Figure 3C). All eight variants resulted in a reduced pumping frequency, with the V84D allele again having the strongest effect (Figure 3B). In theory, a reduction in the overall rate of pharyngeal contractions could be produced either by less frequent yet still rhythmic pumping, or by more arrhythmic pumping. It appears that the latter is the case for the variants, as can be seen in the typical traces in Figure 3A, where periods of rhythmic, regular contractions similar to those seen with wild‐type control are interspersed with bouts of inactivity. Analysis of the IPI standard deviation values demonstrates that there is a greatly increased variability of pharyngeal pumping in all the STXBP1 variants (Figure 3C), with some also exhibiting significant changes in pump duration or IPI duration (Figure S4).

**Figure 4:**
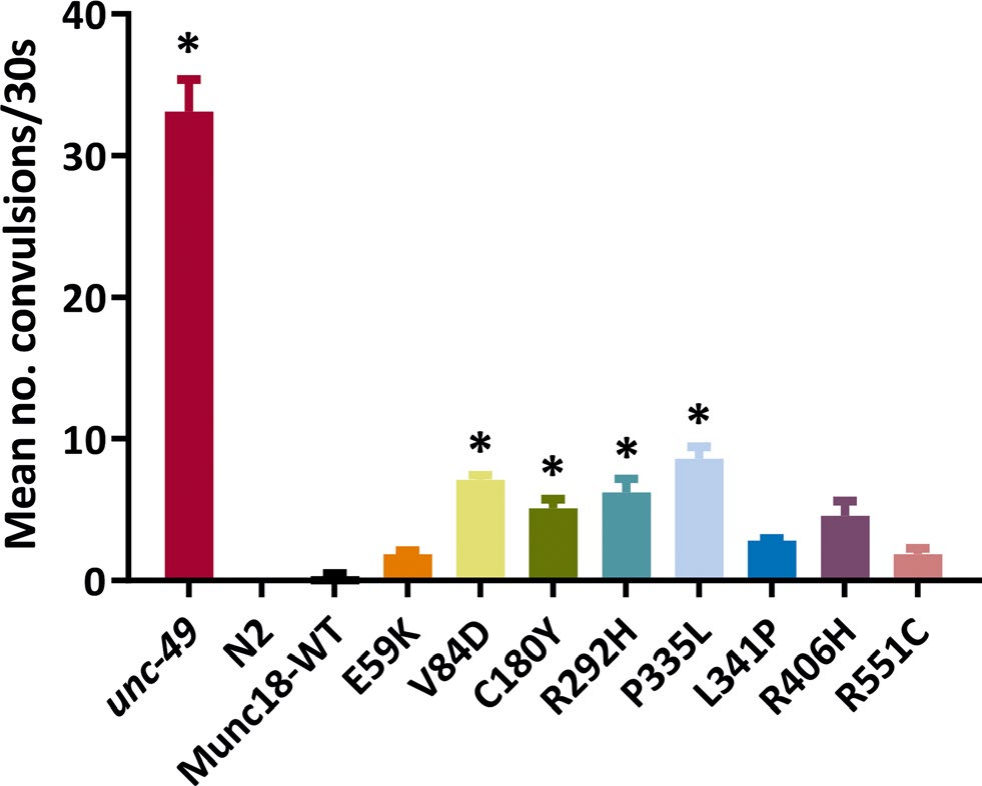
Seizure‐like activity in STXBP1 transgenic worms. Worms were exposed to 7 mg/mL pentylenetetrazol (PTZ) in Dent solution for 15 minutes, and then the number of head‐bobbing convulsions was scored over a 30‐second period. The seizure‐prone *unc‐49* mutant strain was used as a positive control, whereas the wild‐type N2 was used as a negative control, as it does not undergo convulsions in response to PTZ exposure. Four of the variants (V84D, C180Y, R292H, and P335L) displayed significantly higher average convulsions per animal than worms expressing wild‐type STXBP1. Ten animals per strain were analyzed in each experiment, and three independent experiments were performed (n = 30 worms per strain in total). Data are shown as mean ± standard error of the mean (convulsion rates were compared via the Kruskal‐Wallis test with Dunnett multiple comparison correction; *P < .05). Significant *P* values of STXBP1 mutants are as follows: V84D (.000454), C180Y (.019696), R292H (.025307), and P335L (.010462)

To investigate whether the humanized strains displayed seizure‐like activity, we took advantage of a recently developed assay.^31^, ^37^ This involves incubating worms with the proconvulsant PTZ and measuring the number of head‐bobbing convulsions produced. This type of PTZ‐induced convulsion has been well characterized using γ‐aminobutyric acidergic (GABAergic) mutants,^31^, ^38^ and so we included the *unc‐49(e407)* strain harboring a null mutation in a worm GABA_A_ receptor as a positive control. PTZ treatment induced rapid head bobbing in >80% of *unc‐49* worms tested (Video S5), but had no convulsant effect on wild‐type N2 worms, consistent with published findings (Figure 4).^31^, ^37^, ^38^ Humanized worms rescued with wild‐type Munc18‐1 behaved similarly to N2 worms, in that repetitive head bobbing was never observed (Figure 4, Video S6). However, 10%‐30% of the animals expressing STXBP1 variants exhibited seizure‐like activity in response to PTZ. Quantification of the number of head bobs revealed that four of the variants (V84D, C180Y, R292H, P335L) exhibited significantly increased convulsions (Figure 4). Interestingly, the P335L variant strain had the strongest effect in this assay (Figure 4, Video S7), even though it was phenotypically wild type in simple locomotion assays (Figure 2).

Guiberson et al^25^ have recently reported that treatment with chemical chaperones can rescue the phenotype of transgenic *C. elegans* expressing UNC‐18 protein with mutations analogous to several STXBP1 variants. To determine whether we could replicate this finding using our humanized worm strains, we used the identical approach of culturing worms in the presence of 5 mmol/L phenylbutyrate (Pb), 200 mmol/L sorbitol (Sb), or 200 mmol/L trehalose (Tr). We chose five strains to analyze in body bend assays (wild‐type Munc18‐1, V84D, C180Y, R292H, P335L), as these ranged from the most severe (V84D) to no effect (P335L) and as locomotion is more suitable for high‐throughput drug screening than electrophysiological and seizure assays. The reduced locomotion of C180Y was increased by both Pb and Sb, whereas R292H locomotion was increased by Sb only (Supplementary Figure S5). No effect was observed in transgenic worms expressing wild‐type Munc18‐1 or P335L, which exhibit no locomotion defect, indicating that the observed stimulation by Pb and Sb was not due to a general increase in body bends. The most severe mutant, V84D, was also unaffected by any of the three compounds, suggesting that chemical chaperones have therapeutic potential for some, but not all, STXBP1 variants.

### 3.3 Effect of STXBP1/Munc18‐1 variants on mRNA and protein expression

**Figure 5:**
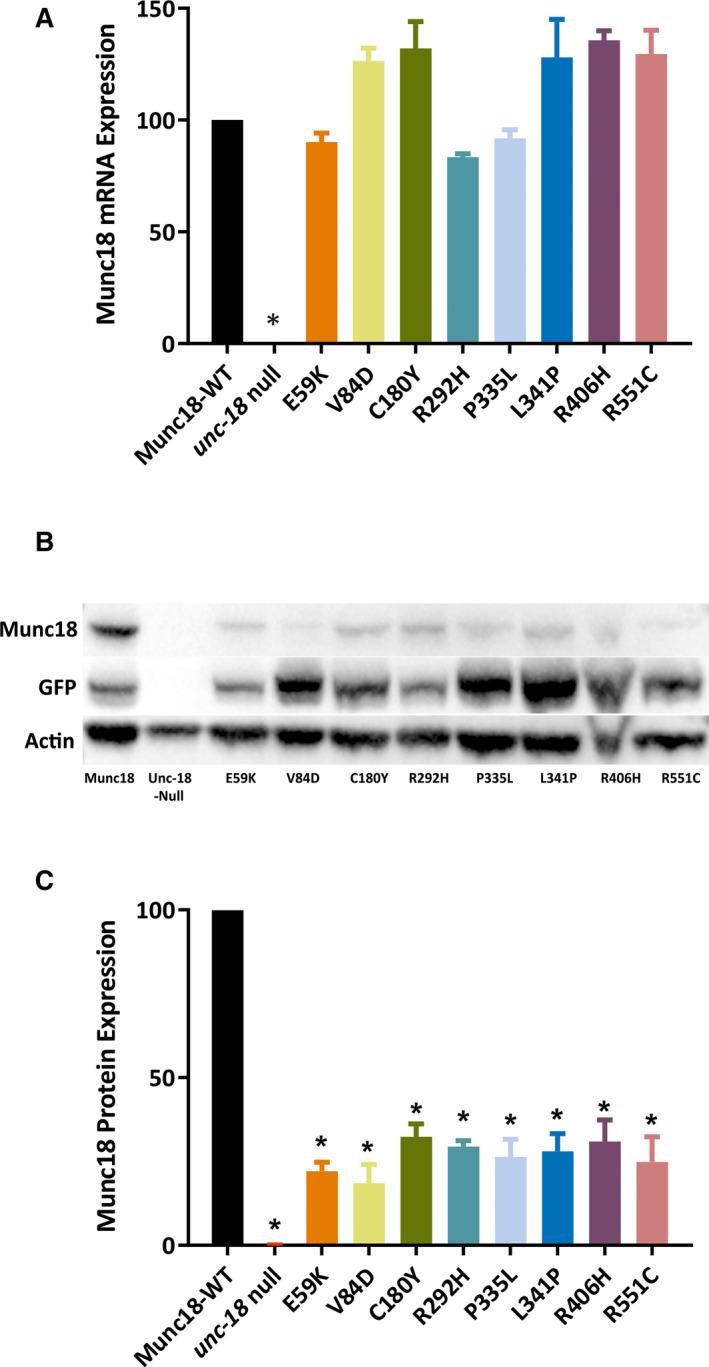
Detection of STXBP1 (Munc18‐1) expression in transgenic worms. A, Transcription of *STXBP1* in the transgenic worm strains was measured by quantitative polymerase chain reaction. None of the mutants’ mRNA level was significantly different from the wild‐type *STXBP1* control. B, Representative Western blots showing that STXBP1 (Munc18‐1) protein expression levels of all mutants are decreased compared to the wild‐type control. The top row shows the result using a Munc18‐1 antibody; the middle row is a green fluorescent protein (GFP) loading control, as GFP has been used as the transgene selection marker; and the beta‐actin protein loading control is shown in the bottom row. C, Based on densitometric quantification of STXBP1 protein expression normalized to GFP, all mutants exhibited significantly reduced STXBP1 protein levels compared to the wild‐type rescue control. Data are shown as mean ± standard error of the mean of three independent experiments. Statistical analysis was performed using one‐way analysis of variance with Tukey correction for multiple comparisons; *P < .05. Significant *P* values of STXBP1 mutants are as follows: E59K (.001175), V84D (.004646), C180Y (.003302), R292H (.000630), P335L (.005151), L341P (.005294), R406H (.008460), and R551C (.009779)

One potential explanation for the varied functional effects seen with different *STXBP1* variants is that this simply reflects variability in transgene copy number or transgene expression. To test this, we assayed mRNA levels in the various strains by quantitative reverse transcription PCR (Figure 5A). As expected, *STXBP1* mRNA was undetectable in the untransformed *unc‐18(ulv12)* strain. However, there were no significant differences in *STXBP1* mRNA levels between the wild‐type and variant humanized strains, thus ruling out this trivial explanation. As it has been reported by several groups that posttranslational stability of Munc18‐1 is reduced by epilepsy‐associated missense mutations,^3^, ^21^, ^24^, ^25^ we performed Western blotting on worm extracts from the humanized strains. It was evident that all eight variants reduced the level of Munc18‐1 protein compared to the wild‐type control strain (Figure 5B). Quantification of protein abundance revealed a profound decrease to 20%‐30% of wild‐type Munc18‐1 protein levels (Figure 5C). Notably, there were no significant differences in Munc18‐1 protein expression levels between the different variants, suggesting that the observed phenotypic differences between the humanized strains cannot be explained entirely by altered protein abundance.

## 4 DISCUSSION

In this work, we have shown using quantitative behavioral and electrophysiological analyses that the function of the *C. elegans* UNC‐18 protein can be fully complemented by transgenic expression of human STXBP1/Munc18‐1 protein. This confirmed and extended the original observation by Gengyo‐Ando et al,^28^ enabling us to test the functional impact of eight epilepsy‐associated variants. All eight were found to be deleterious, as they resulted in decreased STXBP1/Munc18‐1 protein abundance and in less frequent and more irregular pharyngeal muscle contraction. This therefore validates the prediction by SIFT that the E59K is deleterious, in contrast to the benign prediction made by PolyPhen‐2.^10^ However, the effects of the eight variants varied considerably between different functional assays. For example, the P335L strain was indistinguishable from wild‐type in locomotion assays, yet exhibited strong pharyngeal and seizure phenotypes. The reason for this is not clear, but we speculate that it may reflect differential demands on synaptic vesicle exocytosis, as described below.

Serotonin regulation of pharyngeal pumping requires high‐frequency, high‐fidelity synaptic transmission via the two MC neurons to achieve precise rhythmic muscle contraction at around 4 Hz,^36^ and so may be more sensitive to partial inhibition of STXBP1. In contrast, locomotion involves the concerted action of dozens of motor neurons, each making multiple en passant synaptic contacts with muscle arms, and so may have more intrinsic buffering capacity. In addition, the functional impact of STXBP1 variants on synaptic exocytosis may differ between neuronal cell types. For example, GABAergic synapses exhibit a much higher level of spontaneous neurotransmitter release than cholinergic synapses in *C. elegans*, an effect that is blocked in *unc‐18* mutants.^39^ Furthermore, the expression level of molecular chaperone proteins is likely to differ between neurons, which could differentially affect the misfolding/aggregation of STXBP1 variants. As the worm seizure assay relies on reducing GABAergic activity using PTZ, individual STXBP1 variants may selectively affect seizure phenotypes depending on the extent to which they affect GABAergic versus cholinergic signaling and on the functional impact of coexpressed chaperones. It may be of interest in the future to test our humanized strains in other *C. elegans* behavioral assays, including heat‐induced^40^ and electroshock^41^ seizure assays. Regardless of the precise mechanisms involved, our data clearly show that it is best to use a battery of functional assays, rather than a single readout, when testing the functional effect of genetic variants.

Four of the variants studied here (E59K, R292H, L341P, and R551C) have not previously been tested for pathogenicity in cell culture or animal models. However, the four remaining missense variants (V84D, C180Y, P335L, and R406H) have been analyzed before in one or more models, enabling comparison with our findings using humanized worms. All four variants have been shown to cause protein instability, based on studies using recombinant proteins (C180Y),^1^, ^21^ neuronal model cell lines (V84D, C180Y),^4^, ^21^, ^23^ primary cultured neurons (V84D, C180Y, P335L, R406H),^24^, ^25^ or *C. elegans* containing mutations in the homologous UNC‐18 residues (P334L, R405H).^25^ That we find the same here validates our humanized worm approach and suggests that most disease‐causing mutations destabilize the STXBP1/Munc18‐1 protein. This is consistent with the view that all *STXBP1* epilepsy‐associated mutations essentially result in haploinsufficiency.^3^, ^4^, ^24^

Although haploinsufficiency is clearly important, work published over the past few years has suggested an alternative pathogenic mechanism by which missense *STXBP1* mutations may act.^42^ It has been found that some aggregation‐prone mutant proteins can act as a template for wild‐type Munc18‐1 to polymerize, thereby seeding coaggregation and hence acting in a dominant negative manner.^23^, ^25^ Furthermore, STXBP1 mutant proteins have been shown to interact with and seed coaggregation of other neuronal proteins, such as α‐synuclein.^23^ This in turn has therapeutic implications, as re‐establishing expression levels of wild‐type Munc18‐1 may not be able to rescue the underlying defect in such cases. The templating mechanism could explain why some variants have stronger phenotypes than others in our humanized worms, despite being expressed at the same level, if one assumes that the mutant Munc18‐1 seeds coaggregation with endogenous *C. elegans* synaptic proteins. Furthermore, in studies predating the identification of E59K as an epilepsy‐associated variant, this mutation was shown to strongly impair SNARE complex binding in addition to causing Munc18‐1 protein instability^43^, ^44^ (although the reported functional consequence of the SNARE complex effect^43^ was contested^44^). Similarly, studies of a P335A mutation comparable to the P335L variant has established that mutation at this residue confers a gain‐of‐function phenotype that enhances Munc18‐1’s stimulatory effects on SNARE complex assembly and exocytosis.^45^, ^46^, ^47^, ^48^ Interestingly, the V84D allele, which we found to be the most deleterious in humanized *C. elegans* and could not be rescued by chemical chaperones, was the only missense mutation that was unable to rescue neuronal viability in primary neurons derived from *stxbp1* knockout mice.^24^ Therefore, although haploinsufficiency is undoubtedly the cause of epilepsy for many *STXBP1* mutations, the differential severity of some variants suggests that additional functional defects and a capacity to template coaggregation of wild‐type Munc18‐1 with other synaptic proteins may be relevant in some cases.

In summary, the humanized worm approach we describe here provides a cheap, relatively rapid method for establishing the functional impact of *STXBP1* missense variants in living animals. This has relevance not only for epilepsy, but also for intellectual disability without epilepsy, as both conditions are associated with *STXBP1* variants.^10^ In addition, the humanized worm strains could be a useful resource for future drug screens to identify novel therapies. Guiberson et al^25^ have recently reported that chemical chaperones can ameliorate the phenotypic effects of UNC‐18/STXBP1 mutations, which we have confirmed using our humanized strains (Figure S5). Finally, the general strategy described here could potentially be applied to other neurological disorders caused by mutations in genes with *C. elegans* orthologues.

## CONFLICT OF INTEREST

None of the authors has any conflict of interest to disclose. We confirm that we have read the Journal's position on issues involved in ethical publication and affirm that this report is consistent with those guidelines.

## AUTHOR CONTRIBUTIONS

B.Z. performed experiments and analyzed data; J.C.H.M. created the *unc‐18(ulv12)* worm strain; A.M., G.J.S., J.W.B., A.G.M., and A.P.M. conceived the study; B.Z., A.P.M., J.W.B., G.J.S., and A.M. interpreted the data; A.M. and B.Z. wrote the manuscript with input from all authors.
