# Supplementary material for: Functional analysis of epilepsy‐associated variants in STXBP1/Munc18‐1 using humanized Caenorhabditis elegans
Source: Epilepsia. 2020 Feb 29;61(4):810–21. doi: 10.1111/epi.16464 (PMC8614121; doi:10.1111/epi.16464)
Supplement: Supplementary file 1 [file EPI-61-810-s007.pdf]

A

ATGTTCTCAT CGTTGACACCCTAGCCATG ***CGG***ATGTTGTCATCCTGCTGCAA **wild-type *unc-18***

ATGTTCTCAT CGTTGACA - - - - - - - - CATG***CGG***ATGTTGTCATCCTGCTGCAA ***unc-18* (*ulv 12*)**

B

Wild type *unc-18*

Met S L K Q I V G H K L L N D V I R P L K K G D G R S A W N V L I V D T L A **Met** R **Met** L S S C C
K **Met** H N I **Met** E E G I T I V E D L N K R R E P L P T L E A I Y L I A P T A E S I D K L I Q D Y C A
R N L Y K C A H V F F T E A C S D Q L F S T L S K S A A A R F I K T L K E I N I A F T P Y E S Q V
F N L D S P D T F F L Y Y N A Q K Q G G L T S N L E R I A E Q I A T V C A T L G E Y P S L R Y R
A D F E R N V E L G H L V E Q K L D A Y K A D D P S **Met** G E G A D K A R S Q L I I I D R G Y D A
I T P L L H E L T L Q A **Met** C Y D L L G I E N D V Y K Y E T G G S D E N L E K E V L L D E N D D L
W V E **Met** R H K H I A V V S Q E V T K N L K K F S E S K G N K G T **Met** D S K S I K D L S **Met** L I
K R **Met** P Q H K K E L N K F S T H I S L A E E C **Met** K Q Y Q Q G V D K L C K V E Q D L S T G I
D A E G E R V R D A **Met** K L **Met** V P L L I D P A V R C E D R L R L I L L Y I L S K N G I T D E N L
N K L L Q H A N I S **Met** A D K E T I T N A A Y L G L N I V T D T G R K K T W T P T K K E R P H E
Q V Y Q S S R W V P V I K D I I E D A I D E R L D T K H F P F L A G R Q V N Q G Y R A P A S A R
Y G Q W H K E R G Q Q S N Y R S G P R L I I Y I I G G V T F S E **Met** R A C Y E V T A A R K P W
E V V I G S D R I I T P D K F L T N L R D L N K P R D I **Stop**

unc-18 (ulv 12)

Met S L K Q I V G H K L L N D V I R P L K K G D G R S A W N V L I V D T C G C C H P A A K C T I
L W K K E L Q L W K I **Stop**

Figure S1. *unc-18 (ulv12)* deletion allele generated using CRISPR/Cas9.
A, DNA sequence showing the 7bp deletion in *unc-18 (ulv12)*. Highlighted blue areas represents the sgRNA targeting sequence used; and red identifies the protospacer adjacent motif.
B, predicted amino acid sequence of translated wild type and mutant UNC-18 protein. Underlined residues indicate altered amino acids and the premature stop codon caused by the frameshift mutation.

```
Munc-18   4   IGLKAVVGEKIMHDVIKKVKK---KGEWKVLVVDQLSMRMLSSCCKMTDIMTEGITIVED   60
              + LK +VG K+++DVI+ +KK   +  W VL+VD L+MRMLSSCCKM +IM EGITIVED
Unc-18    1   MSLKQIVGHKLLNDVIRPLKKGDGRSAWNVLIVDTLAMRMLSSCCKMHNIMEEGITIVED   60
Munc-18  61   INKRREPLPSLEAVYLITPSEKSVHSLISDFKDPPTAKYRRAHVFFTDSCPDALFNELVK  120
              +NKRREPLP+LEA+YLI P+ +S+  LI D+       Y+ AHVFFT++C D LF+ L K
Unc-18   61   LNKRREPLPTLEAIYLIAPTAESIDKLIQDY--CARNLYKCAHVFFTEACSDQLFSTLSK  118
Munc-18 121   SRAAKVIKTLTEINIAFLPYESQVYSLDSADSFQSFYSPHKAQMKNPILERLAEQIATLC  180
              S AA+ IKTL EINIAF PYESQV++LDS D+F  +Y+  K        LER+AEQIAT+C
Unc-18  119   SAAARFIKTLKEINIAFTPYESQVFNLDSPDTFFLYYNAQKQGGLTSNLERIAEQIATVC  178
Munc-18 181   ATLKEYPAVRYRGEYKDNALLAQLIQDKLDAYKADDPTMGEGPDKARSQLLILDRGFDPS  240
              ATL EYP++RYR +++ N  L  L++ KLDAYKADDP+MGEG DKARSQL+I+DRG+D
Unc-18  179   ATLGEYPSLRYRADFERNVELGHLVEQKLDAYKADDPSMGEGADKARSQLIIIDRGYDAI  238
Munc-18 241   SPVLHELTFQAMSYDLLPIENDVYKYETSGIGEARVKEVLLDEDDDLWIALRHKHIAEVS  300
              +P+LHELT QAM YDLL IENDVYKYET G  E   KEVLLDE+DDLW+ +RHKHIA VS
Unc-18  239   TPLLHELTLQAMCYDLLGIENDVYKYETGGSDENLEKEVLLDENDDLWVEMRHKHIAVVS  298
Munc-18 301   QEVTRSLKDFSSSK-RMNTGEKTTMRDLSQMLKKMPQYQKELSKYSTHLHLAEDCMKHYQ  359
              QEVT++LK FS SK    T +  +++DLS ++K+MPQ++KEL+K+STH+ LAE+CMK YQ
Unc-18  299   QEVTKNLKKFSESKGNKGTMDSKSIKDLSMLIKRMPQHKKELNKFSTHISLAEECMKQYQ  358
Munc-18 360   GTVDKLCRVEQDLAMGTDAEGEKIKDPMRAIVPILLDANVSTYDKIRIILLYIFLKNGIT  419
                VDKLC+VEQDL+ G DAEGE+++D M+ +VP+L+D  V   D++R+ILLYI  KNGIT
Unc-18  359   QGVDKLCKVEQDLSTGIDAEGERVRDAMKLMVPLLIDPAVRCEDRLRLILLYILSKNGIT  418
Munc-18 420   EENLNKLIQHAQIPPEDSEIITNMAHLGVPIVTDSTLRRRSKPERKERISEQTYQLSRWT  479
              +ENLNKL+QHA I   D E ITN A+LG+ IVTD+  ++    P +KER  EQ YQ SRW
Unc-18  419   DENLNKLLQHANISMADKETITNAAYLGLNIVTDTGRKKTWTPTKKERPHEQVYQSSRWV  478
Munc-18 480   PIIKDIMEDTIEDKLDTKHYPYISTRSSASFSTTAVSARYGHWHKNKA-PGEYRSGPRFI  538
              P+IKDI+ED I+++LDTKH+P+++ R           SARYG WHK +     YRSGPR I
Unc-18  479   PVIKDIIEDAIDERLDTKHFPFLAGRQVNQGYRAPASARYGQWHKERGQQSNYRSGPRLI  538
Munc-18 539   IFILGGVSLNEMRCAYEVTQANAKWEVLIGSTHILTPQKLLDTLKKLNK  587
              I+I+GGV+ +EMR  YEVT A   WEV+IGS  I+TP K L  L+ LNK
Unc-18  539   IYIIGGVTFSEMRACYEVTAARKPWEVVIGSDRIITPDKFLTNLRDLNK  587
```

Figure S2. Amino acid sequence alignment between human Munc18-1/STXBP1 and *C. elegans* UNC-18 proteins.

Highlighted residues indicate mutations analysed in this study.

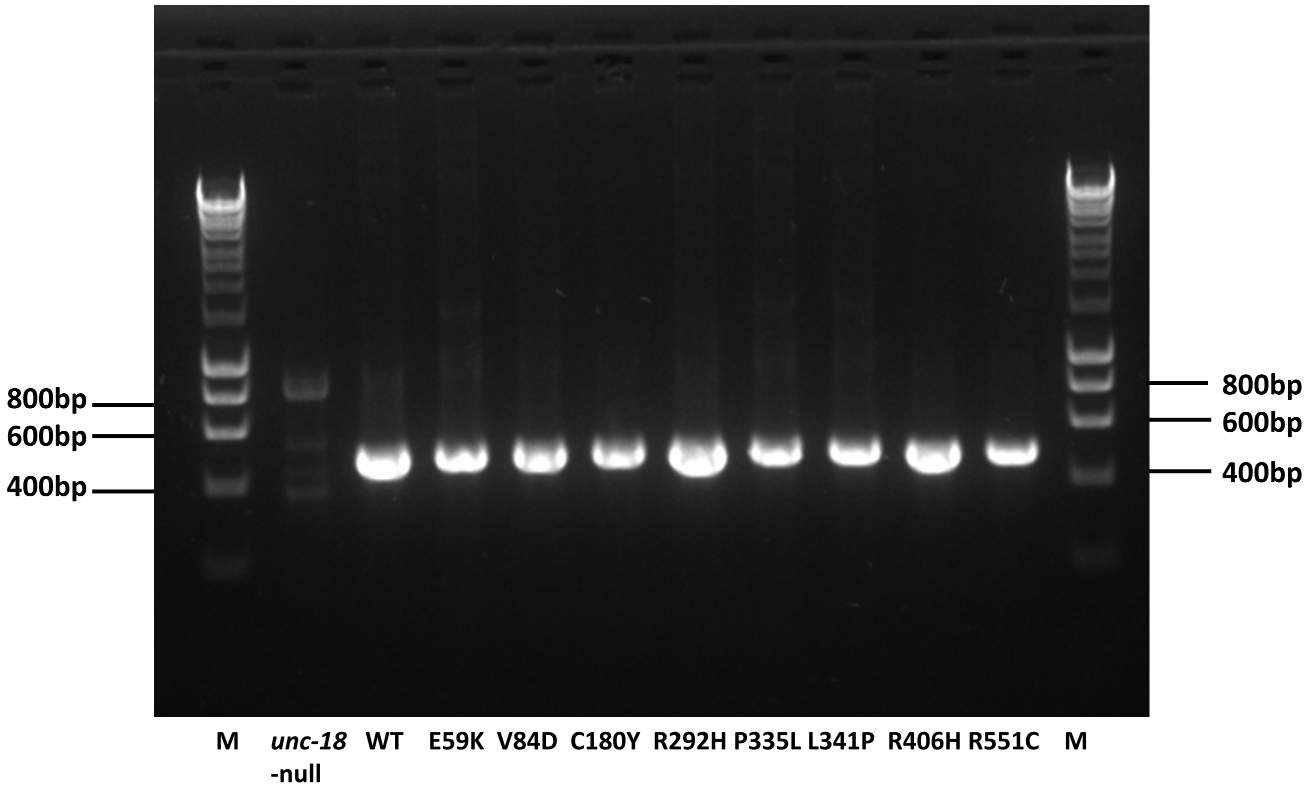

Figure S3. Endpoint PCR confirmation that transgenic *C. elegans* contain *STXBP1*(Munc18-1).

A 500bp DNA fragment was amplified from all transgenic worm strains including Munc18 wild type control (WT) and 8 mutants but not from *unc-18* null mutant (2nd lane) using primers specific to mammalian *STXBP1* cDNA, indicating that gene transfer (by microinjection) was successful. M represents DNA ladders on both sides.

	Mean Frequency (Hz)	Mean Pump Duration (ms)	Mean IPI Duration (ms)	IPI Duration Standard Deviation (ms)
N2	3.93	108.0	215.6	92.4*(p=0.0224)
unc-18 null	0.21*(p=0.0001)	210.13*(p=0.0021)	1025.91*(p=0.0001)	803.53*(p=0.0001)
Munc18-WT	3.49	112.92	271.77	143.58
E59K	2.37*(p=0.0495)	146.69	509.8*(p=0.0463)	330.1*(p=0.0399)
V84D	1.21*(p=0.0002)	151.09*(p=0.0106)	720.98*(p=0.0057)	708.85*(p=0.0143)
C180Y	1.4*(p=0.0003)	203.62*(p=0.0238)	620.9*(p=0.0132)	502.53*(p=0.0458)
R292H	1.37*(p=0.0002)	203.84*(p=0.0374)	600.36*(p=0.0128)	488.26*(p=0.0120)
P335L	1.79*(p=0.0048)	156.34*(p=0.0090)	539.28*(p=0.0250)	398.12*(p=0.0209)
L341P	1.41*(p=0.0003)	221.68*(p=0.0015)	649.99*(p=0.0408)	462.18*(p=0.0174)
R406H	1.84*(p=0.0317)	164.05*(p=0.0313)	457.06	353.17*(p=0.0230)
R551C	2.57*(p=0.0391)	129.0	398.0	309.03*(p=0.0418)

Figure S4. EPG recording parameters from STXBP1 (Munc18-1) transgenic worm strains and wild type Bristol N2 control. Asterisk* represents statistically significant.

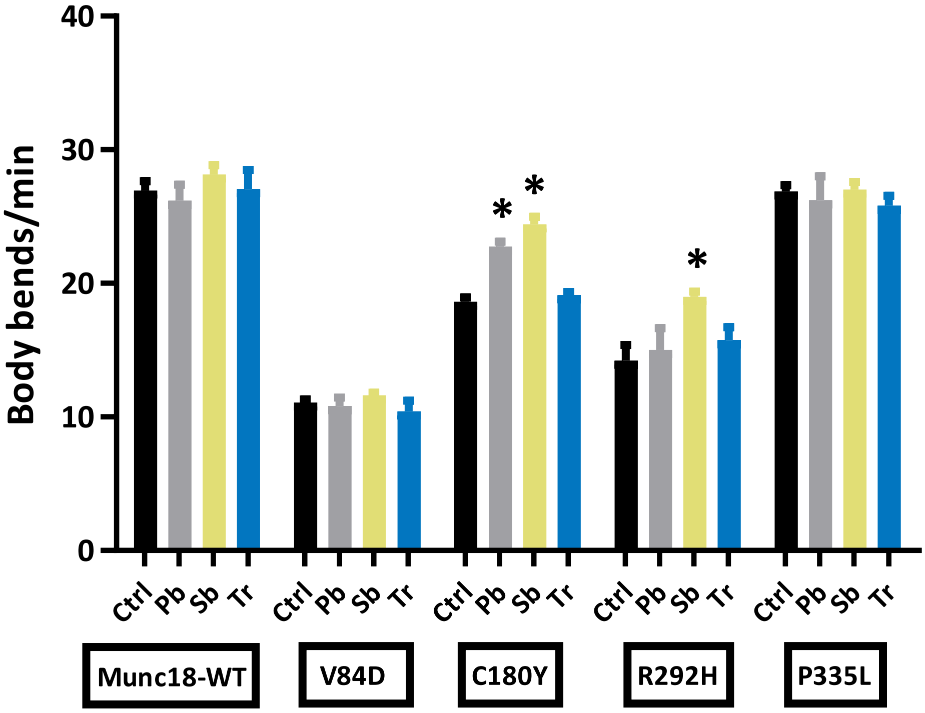

Figure S5. Chemical chaperones ameliorate the locomotion defect of C180Y and R292H STXBP1 transgenic *C. elegans*. Worms were cultured for two-three generations on OP50-seeded NGM agar plates containing either 5 mM 4-phenylbutyrate (Pb), 200 mM sorbitol (Sb), or 200 mM trehalose (Tr). Young adult worms were then subjected to body bend assays on freshly made unseeded NGM plates with each chemical at the concentration described above. The reduced locomotion of C180Y and R292H mutants was significantly increased by at least one of the chemical chaperones. Ten animals per strain were analysed in each experiment, and three independent experiments were performed (n = 30 worms per strain in total). Data are shown as mean ± SEM and statistical analysis was performed using one-way ANOVA with Tukey's correction for multiple comparisons (*P<0.05).
